# *RAG1* co‐expression signature identifies ETV6‐RUNX1‐like B‐cell precursor acute lymphoblastic leukemia in children

**DOI:** 10.1002/cam4.3928

**Published:** 2021-05-13

**Authors:** Dongfeng Chen, Alessandro Camponeschi, Jessica Nordlund, Yanara Marincevic‐Zuniga, Jonas Abrahamsson, Gudmar Lönnerholm, Linda Fogelstrand, Inga‐Lill Mårtensson

**Affiliations:** ^1^ Institute of Life Sciences Jiangsu University Zhenjiang China; ^2^ Department of Rheumatology and Inflammation Research Institute of Medicine Sahlgrenska Academy University of Gothenburg Gothenburg Sweden; ^3^ Department of Medical Sciences Molecular Medicine and Science for Life Laboratory Uppsala University Uppsala Sweden; ^4^ Department of Pediatrics Institute of Clinical Sciences Sahlgrenska University Hospital Gothenburg Sweden; ^5^ Department of Women and Children’s Health Uppsala University Uppsala Sweden; ^6^ Department of Clinical Chemistry Sahlgrenska University Hospital Gothenburg Sweden; ^7^ Department of Clinical Chemistry and Transfusion Medicine University of Gothenburg Gothenburg Sweden

**Keywords:** BCP‐ALL, ETV6‐RUNX1, ETV6‐RUNX1‐like, leukemia, RAG1

## Abstract

B‐cell precursor acute lymphoblastic leukemia (BCP‐ALL) can be classified into subtypes according to the genetic aberrations they display. For instance, the translocation t(12;21)(p13;q22), representing the *ETV6*‐*RUNX1* fusion gene (ER), is present in a quarter of BCP‐ALL cases. However, around 10% of the cases lack classifying chromosomal abnormalities (B‐other). In pediatric ER BCP‐ALL, rearrangement mediated by RAG (recombination‐activating genes) has been proposed as the predominant driver of oncogenic rearrangement. Herein we analyzed almost 1600 pediatric BCP‐ALL samples to determine which subtypes express *RAG*. We demonstrate that *RAG1* mRNA levels are especially high in the ETV6‐RUNX1 (ER) subtype and in a subset of B‐other samples. We also define 31 genes that are co‐expressed with *RAG1* (*RAG1*‐signature) in the ER subtype, a signature that also identifies this subset of B‐other samples. Moreover, this subset also shares leukemia and pro‐B gene expression signatures as well as high levels of the *ETV6* target genes (*BIRC7*, *WBP1L*, *CLIC5*, *ANGPTL2*) with the ER subtype, indicating that these B‐other cases are the recently identified ER‐like subtype. We validated our results in a cohort where ER‐like has been defined, which confirmed expression of the *RAG1*‐signature in this recently described subtype. Taken together, our results demonstrate that the *RAG1*‐signature identifies the ER‐like subtype. As there are no definitive genetic markers to identify this novel subtype, the *RAG1*‐signature represents a means to screen for this leukemia in children.

## Graphical abstract

The recombination‐activating genes (RAG) may play a role in leukemogenesis. We determined, therefore, in around 1600 BCP‐ALL samples which subtypes express RAG and defined a set of co‐expressed genes, the RAG1‐signature, which was thereafter used to identify potential new subtypes. We demonstrate that the RAG1‐signature identifies the recently described ETV6‐RUNX1‐like BCP‐ALL.

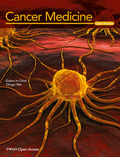

## 1 INTRODUCTION

Acquired chromosomal aberrations have been linked to the overall survival of patients with B‐cell precursor acute lymphoblastic leukemia (BCP‐ALL), which is the most common cancer in children.^1^ The many different subtypes of BCP‐ALL have been classified according to the genetic aberrations they display, allowing correlations between disease type and prognosis to be made.^2^ For instance, the translocation t(12;21)(p13;q22), representing the *ETV6*‐*RUNX1* fusion gene (ER), is present in a quarter of BCP‐ALL cases. Unclassified cases form a heterogeneous group referred to as B‐other, which research efforts have reduced over the last 10 years from 25% to 5% of all cases.^3^ The characterization of disease genotype in such cases remains a priority, as it provides the means for diagnosis, prognosis, risk assessment, and targeted treatment.

During the early stages of B cell development, B‐cell precursors (BCP) undergo immunoglobulin (*Ig*) gene rearrangement that is necessary for the production of a membrane‐bound B‐cell antigen receptor (BCR) on more mature B cells. Mouse knockout models,^4^, ^5^ have shown the recombination‐activating genes (*RAG*) to be essential for the rearrangement process, in which *Ig V(D)J* gene segments are rearranged to provide instructions for a unique BCR. RAG activity may also play a role in leukemogenesis, as has been proposed for the ER subtype, where it appears that RAG introduces mutations and aberrant rearrangements in non‐Ig loci.^6^ However, RAG is likely not responsible for the ER translocation, but rather for introducing additional genetic modifications that drive leukemogenesis. Herein, we examined 1582 BCP‐ALL cases, both microarray (DS1‐6‐M) and RNAseq (DS7‐8‐R) data sets (Table 1, Table S1), to determine on a large scale which subtypes express *RAG* and whether we could find any co‐expressed genes that would allow us to identify new subtypes.

**TABLE 1:** Data sets used in this study

GEO accession	Dataset#	Country	Platform	Patient #	References
Healthy (Fetal BM)					
GSE45460	DS0‐M	South Korea	GPL6244	8	[7]
BCP‐ALL (Pediatric)					
GSE26281	DS1‐M	USA	GPL96	127	[8]
GSE28497	DS2‐M	USA	GPL96	239	[9]
GSE47051	DS3‐M	Sweden	GPL570	75	[10]
GSE12995	DS4‐M	USA	GPL96	175	[11]
GSE33315	DS5‐M	USA	GPL96	483	[12]
GSE26366	DS6‐M	USA	GPL96	172	[13]
RNA‐seq1	DS7‐R	Sweden	Hiseq2000/2500	116	[14]
RNA‐seq2	DS8‐R	Sweden	NextSeq 500	195	[15]
BCP‐ALL (Adult)					
GSE34861	DS9‐M	USA	GPL15088	194	[16]
B95	DS10‐M	USA	GPL8300	95	[17]

## 2 MATERIALS AND METHODS

### 2.1 Gene expression microarray data

Gene expression data from BCP‐ALL and healthy B‐cells were gathered from published studies (Table 1, Table S1). All gene expression microarray data were log2 transformed and normalized using the Robust Multichip Average (RMA) method.

### 2.2 Gene expression RNA‐sequencing data

Strand‐specific RNA sequencing libraries were constructed from rRNA‐depleted RNA using the ScriptSeq V2 Kit (Epicentre) and sequenced paired‐end on a HiSeq or MiSeq sequencer (Illumina Inc). The reads were mapped to the human 1000 Genomes build 37 (GRCh37) using Tophat 2. Gene expression counts were summarized at the exon‐level per gene using feature counts. Additional details can be found in Ref. [7].

### 2.3 Data analysis

All the gene expression microarray and RNA‐seq data were analyzed using Qlucore Omics Explorer 3.5 (Qlucore AB). Genes co‐expressed with *RAG1* were identified by using Pearson's correlation coefficient analysis, and the corr. value was set as 60%. To assess the similarity of molecular signatures between pro‐B cells (pro‐B signature) and ETV6‐RUNX1 or ETV6‐RUNX1 like BCP‐ALL, gene set enrichment analysis (GSEA) was performed. To compare the sample clusters, principal components analysis (PCA) was run. Where needed, data were analyzed by one‐way analysis of variance (ANOVA) or unpaired two‐tailed *t*‐tests using Graph‐Pad Prism version 9, and statistical significance was set as: *p < 0.05, **p < 0.01, ***p < 0.001 and ****p < 0.0001.

## 3 RESULTS AND DISCUSSION

**FIGURE 1:**
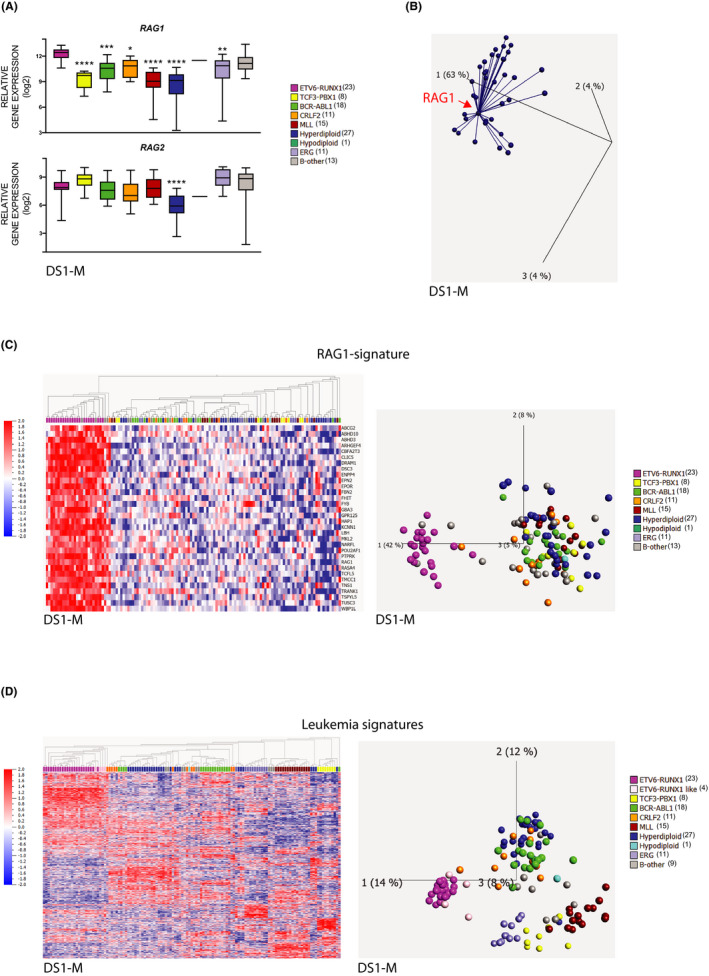
(A) Box plot shows the expression patterns of *RAG1* and *RAG2* in 127 BCP‐ALL samples from DS1‐M (GSE26281), one‐way ANOVA was used as statistical analysis, (*p < 0.05, **p < 0.01, ***p < 0.001, ****p < 0.0001). (B) PCA chart shows that 31 genes co‐expressed with *RAG1* (*RAG1*‐signature) are identified in DS1‐M. Corr Value = 60%, using Pearson's Correlation Coefficient analysis. (C) Heatmap shows the new cluster distribution of samples with different genetic subtypes based on the levels of the *RAG1*‐signature in DS1‐M using hierarchical clustering analysis. PCA plot shows the new cluster pattern based on the *RAG1*‐signature in DS1‐M. Four B‐other and one CRLF2 samples express the *RAG1*‐signature. (D) Unsupervised PCA analyses in DS1‐M show ER and ER‐like BCP‐ALL sharing similar gene expression profiling (*p* = 1.8E‐6, 1240 genes)

We have previously shown that the components of the pre‐BCR complex, assembled from Ig heavy chain and surrogate light chain, are differentially expressed in the ETV6‐RUNX1 and TCF3‐PBX1 BCP‐ALL subtypes.^18^ To determine the expression pattern of *RAG1* and *RAG2* that regulate Ig gene rearrangements, we first analyzed the expression of the *RAG1* and *RAG2* genes in the microarray dataset DS1‐M that was used in the aforementioned study, which includes 127 BCP‐ALL samples (Table 1, Table S1). We found both genes expressed in the ER subtype in this (Figure 1A) and the additional five microarray datasets (DS2‐6‐M) analyzed (Figure S1A). In particular, the expression of *RAG1* was consistently higher in ER compared to all other genetic subtypes except B‐other. Next, we performed a genome‐wide screen for genes co‐expressed with *RAG1* and *RAG2* in DS1‐M using Pearson co‐efficiency correlation analysis. Although none consistently appeared with *RAG2*, we identified a set of 31 genes that were the highest‐ranked co‐expressed genes with *RAG1*, henceforth referred to as the *RAG1*‐signature (Figure 1B, Tables S2 and S3). Using the *RAG1*‐signature as an identifier in the DS1‐M data set distinguished the ER from all other BCP‐ALL subtypes, except for four B‐other and one CRLF2 samples that also expressed the *RAG1*‐signature (Figure 1C). By contrast, although pro‐ and pre‐B cells from healthy donors (DS0‐M, Table 1) express *RAG1* and *RAG2*, they do not express the *RAG1*‐signature (Figure S2), demonstrating that this signature is specific for certain BCP‐ALL.

Based on the above results, we hypothesized that the B‐other samples with a *RAG1*‐signature could represent the recently defined new subtype termed ER‐like,^14^, ^15^, ^19^ which usually carry *ETV6* fusions and *IKZF1* aberrations. However, they vary and hence lack the definitive ER fusion gene, predicting a similar gene expression pattern to the ER subtype. Therefore, to pinpoint the relationships between genetic subtypes, we performed an unsupervised PCA analysis based on all genes expressed in DS1‐M. This showed that the four B‐other samples with the *RAG1*‐signature clustered together with the ER samples with a unique leukemia signature (Figure 1D, labeled ER‐like to distinguish them from the remaining B‐other samples). We could confirm these results by analyzing the other five microarray data sets (DS2‐M to DS6‐M) with a total of 1145 samples, where the *RAG1*‐signature distinguished the ER from the other subtypes, and in each data set a few B‐other samples clustered with the ER samples (Figure S3). Moreover, we found that these samples expressed a leukemia‐signature and clustered with the ER samples also in these microarray data sets (Figure S4).

**FIGURE 2:**
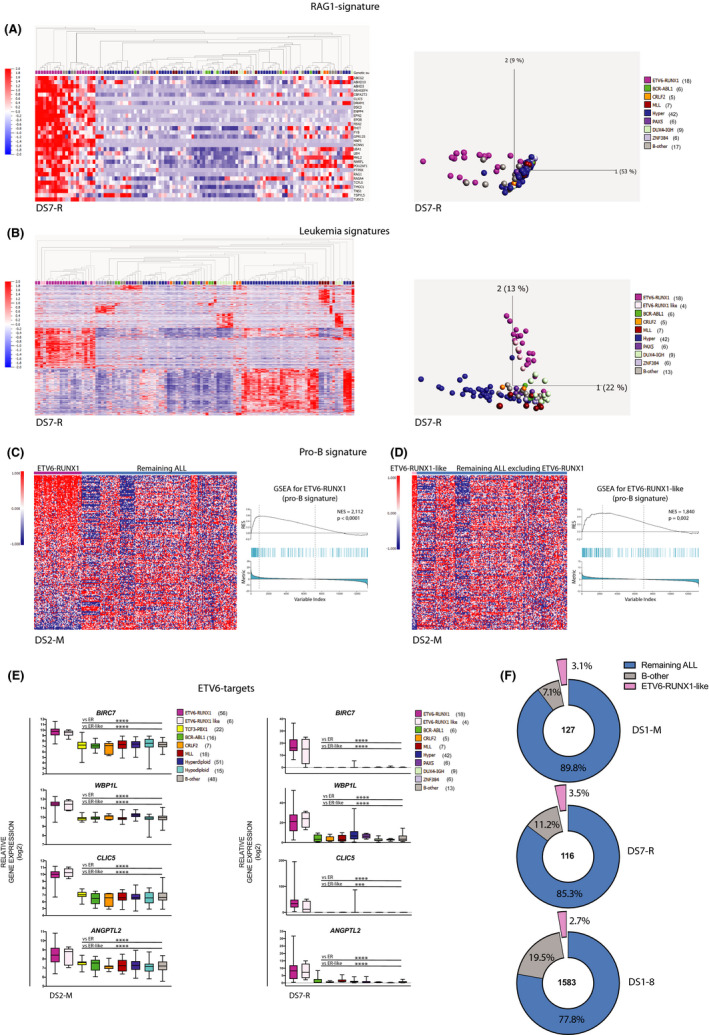
(A) Heatmap and PCA chart show the new cluster pattern based on the *RAG1*‐signature in the validating RNA‐seq data set (*n* = 116, DS7‐R). Four B‐other and one HH patients express the *RAG1*‐signature. (B) Unsupervised PCA analyses in DS7‐R show ER and ER‐like BCP‐ALL sharing similar gene expression profiling (*p* = 2.8E‐6, 1640 genes). Gene Set Enrichment Analyses (GSEA) show the enrichment of the pro‐B signature in (C) ER and (D) ER‐like BCP‐ALL in DS2‐M. (E) Box plots show the expression patterns of *ETV6* target genes in DS2‐M and DS7‐R, unpaired two‐tailed *t*‐tests were used as statistical analysis, (*p < 0.05, **p < 0.01, ***p < 0.001, ****p < 0.0001). (F) Pie charts show ER‐like frequency among BCP‐ALL in DS1‐M, DS7‐R, and in all data sets (DS1‐8). The numbers in the center of the pie charts represent the number of samples. (B) Kaplan–Meier survival analysis was used to estimate the survival of patients in the indicated data sets. Survival in clusters was compared using the log‐rank test

To validate our observations based on microarray datasets, we analyzed RNA‐seq data from a cohort of 116 BCP‐ALL samples (DS7‐R). Here we found not only *RAG1* but also *RAG2* expressed at higher levels in the ER compared to the other subtypes (Figure S1B). Moreover, as in the microarray data sets, a few samples belonging to the B‐other group also expressed higher levels of *RAG1* and *RAG2*. Further, using the *RAG1*‐signature as an identifier in this RNAseq data set distinguished the ER from the other subtypes (Figure 2A and Table S3). We found also four B‐other and one hyperdiploid (HH) samples that clustered with the ER samples (Figure 2A). This would be consistent with our previous analysis of the DNA methylation pattern of the patient samples in DS7‐R in which three of the four samples identified here showed a pattern similar to that of the ER samples.^20^ Thereafter, we performed unsupervised PCA analyses based on gene expression, which confirmed that the same B‐other samples (labeled ER‐like) clustered with the ER samples (Figure 2B). These results support the notion that the B‐other samples expressing the *RAG1*‐signature represent the ER‐like subtype. To gain further support for this hypothesis, we analyzed one more RNAseq data set comprising 195 samples (DS8‐R) in which the ER‐like subtype was recently identified.^15^ Also in this dataset did we find the *RAG1*‐signature in the ER samples as well as in those defined as ER‐like (Figure S5). Taken together, we conclude that the *RAG1*‐signature identifies both the ER and the ER‐like subtypes.

We have previously shown that common lymphoid progenitors (CLP), pro‐B, pre‐B, and immature B cells from healthy donors display unique gene expression signatures.^18^, ^21^ Moreover, the ER subtype displays a pro‐B signature, whereas the t(1;19) TCF3/PBX1 ALL subtype resembles pre‐B cells.^18^ Here, we confirmed the pro‐B signature in ER samples in DS2‐M with 239 BCP‐ALL samples (Figure 2C). Considering its similarities to the ER subtype, we asked whether also ER‐like patient samples displayed the pro‐B signature. To reduce the dominant effect of the ER samples, they were excluded from this analysis. Our analyses showed that the pro‐B signature was present in the ER‐like samples as well (Figure 2D), an observation we could confirm in the two additional datasets analyzed (Figure S6).

*ETV6* encodes a transcription factor that suppresses the expression of genes such as *WBP1L* and *CLIC5*,^22^ which are both found in the *RAG1*‐signature. In the ER subtype, *ETV6* is translocated on one allele and in some samples deleted on the other, resulting in a dysfunctional protein and/or reduced levels. Moreover, the HH patient sample in DS7‐R that clustered with the ER samples (Figure 2A) harbors a t(7;12) *CBX3*‐*ETV6* fusion gene supporting the ER‐like phenotype.^20^ Also, the ER‐like samples in DS8‐R have been found to harbor deletions and in‐frame fusions that involve *ETV6*.^15^ We hypothesized, therefore, that aberrant levels or a dysfunctional *ETV6*, might result in elevated levels of its target genes. We asked, therefore, whether its target genes were expressed in the ER and ER‐like samples in both the microarray and RNAseq data sets. Consistent with the aforementioned notion, the expression levels of the *ETV6*‐target genes *WBP1L, CLIC5, BIRC7*, and *ANGPTL2* were expressed at very high levels in both ER and ER‐like samples, with a consistent pattern and levels not observed in any of the other subtypes (Figure 2E and Figure S7). Thus, gaining further support that also the ER‐like subtype is deficient in the ETV6 transcription factor.

Our results suggest that the ER‐like subtype is infrequent among BCP‐ALL. Analyzing the frequency showed that it was 3.1% in DS1‐M, 3.5% in DS7‐R, and taken all data sets (DS1‐8) together, hence 1582 BCP‐ALL samples, demonstrated that an average of 2.7% correspond to the ER‐like subtype (Figure 2F, Table S4).

In adults BCP‐ALL of the ER subtype are infrequent. We asked therefore whether we could find any ER‐like samples based on the *RAG1*‐signature. However, among a total of 285 samples (DS9‐10‐M) with only one ER sample (Table 1, Table S1), which we could clearly distinguish, we were unable to define any ER‐like samples (Figure S8). Thus, this indicates that not only ER but also ER‐like subtypes are infrequent in adult BCP‐ALL.

In this study, we show that a subset of BCP‐ALL patient samples with unclassified chromosomal abnormalities (B‐other) can be defined by the expression of *RAG1* in conjunction with an additional 31 genes, the *RAG1*‐signature. This signature as well as leukemia and pro‐B gene expression signatures and the high levels of *ETV6* target genes were all shared with the ER subtype, and suggested that these B‐other cases belong to the ER‐like subtype. Validating this in samples previously defined as ER‐like, we could confirm this notion. Taken together, our results demonstrate that the *RAG1*‐signature identifies ER‐like BCP‐ALL in children. As there are no consistent translocations or other definitive genetic markers, using the *RAG1*‐signature could represent a means to screen for the ER‐like subtype.

## CONFLICT OF INTEREST

The authors declare no conflict of interest.

## ETHICAL CONSIDERATIONS

Not applicable, meta‐analyses, all ethics linked to respective data set, see Table 1.

## Supporting information

Figure S1

Figure S2

Figure S3

Figure S4

Figure S5

Figure S6

Figure S7

Figure S8

Table S1‐S2 and S4

Table S3

## Data Availability

Gene expression data from BCP‐ALL and healthy B‐cells were gathered from published studies (Table 1).
