# Supplementary figures and images for: RAG1 co‐expression signature identifies ETV6‐RUNX1‐like B‐cell precursor acute lymphoblastic leukemia in children
Source: Cancer Med. 2021 May 13;10(12):3997–4003. doi: 10.1002/cam4.3928 (PMC8209579; doi:10.1002/cam4.3928)

FIGURE S1

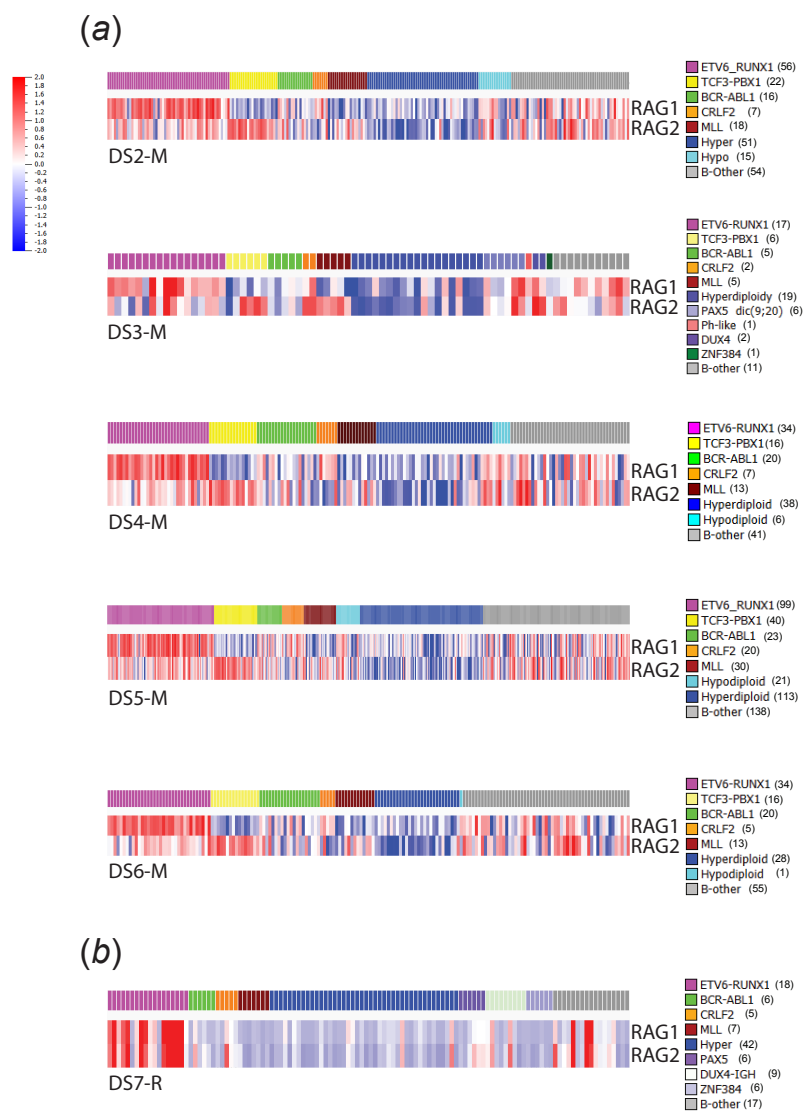

Figure S1. Heatmap shows the expression patterns of *RAG1* and *RAG2* in (a) DS2-M to DS6-M and (b) DS7-R.

Supplement: Supplementary file 1 — Figure S1 [file CAM4-10-3997-s004.pdf]

Figure S7

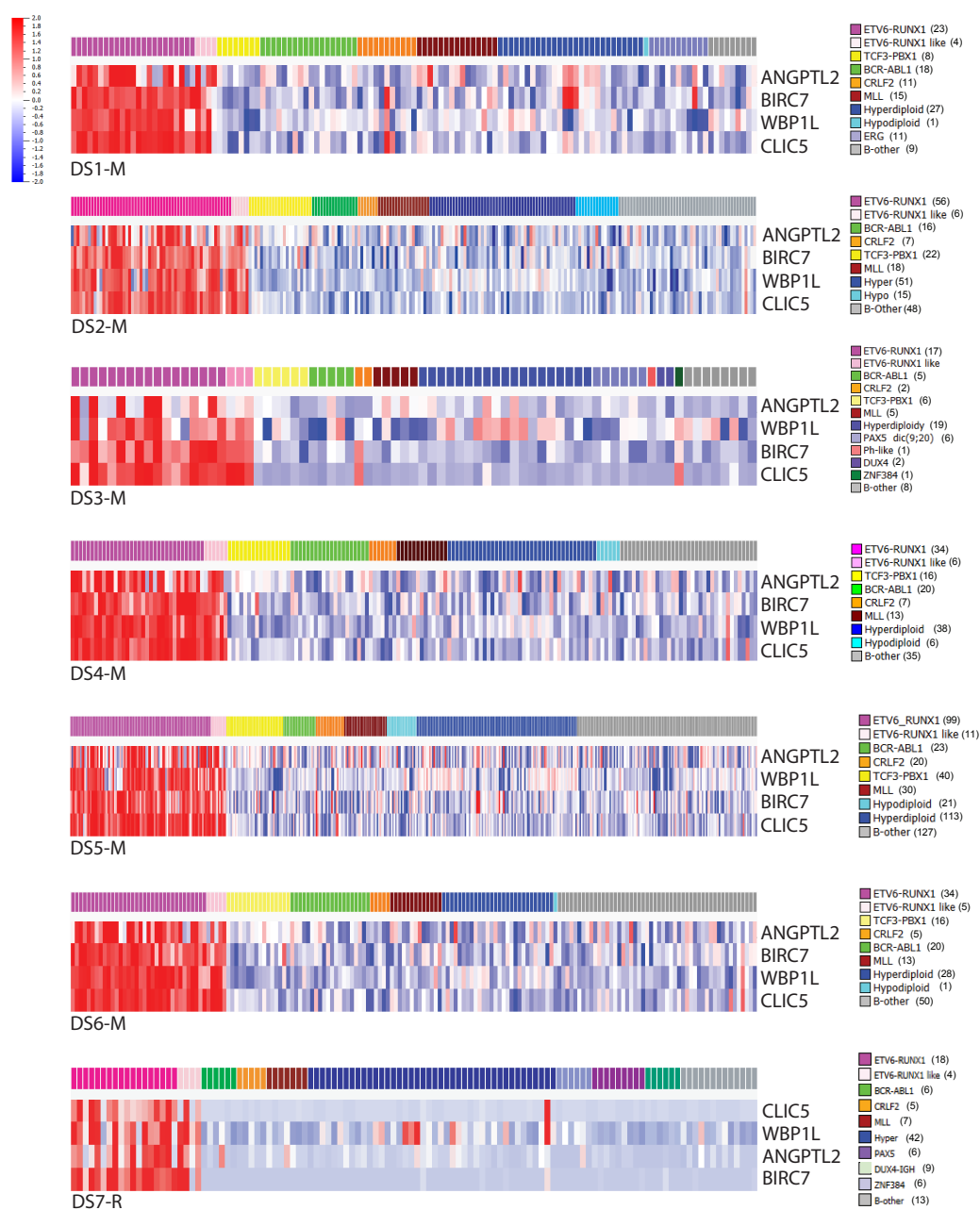

Figure S7. Heatmaps show the expression patterns of *ETV6* target genes in DS1-6-M and DS7-R.

Supplement: Supplementary file 7 — Figure S7 [file CAM4-10-3997-s010.pdf]
