# Supplementary material for: RAG1 co‐expression signature identifies ETV6‐RUNX1‐like B‐cell precursor acute lymphoblastic leukemia in children
Source: Cancer Med. 2021 May 13;10(12):3997–4003. doi: 10.1002/cam4.3928 (PMC8209579; doi:10.1002/cam4.3928)
Supplement: Supplementary file 2 — Figure S2 [file CAM4-10-3997-s008.pdf]

Figure S2

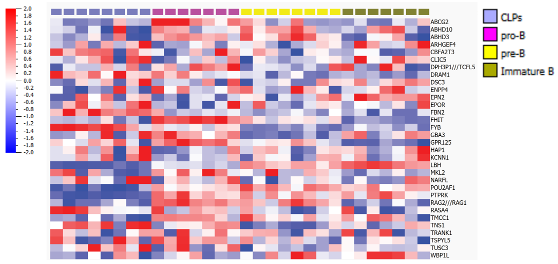

DS0-M

Figure S2. Heatmap shows the expression pattern of RAG1-signature genes in healthy B cell data set DS0-M.
