# Supplementary material for: RAG1 co‐expression signature identifies ETV6‐RUNX1‐like B‐cell precursor acute lymphoblastic leukemia in children
Source: Cancer Med. 2021 May 13;10(12):3997–4003. doi: 10.1002/cam4.3928 (PMC8209579; doi:10.1002/cam4.3928)
Supplement: Supplementary file 3 — Figure S3 [file CAM4-10-3997-s005.pdf]

Figure S3

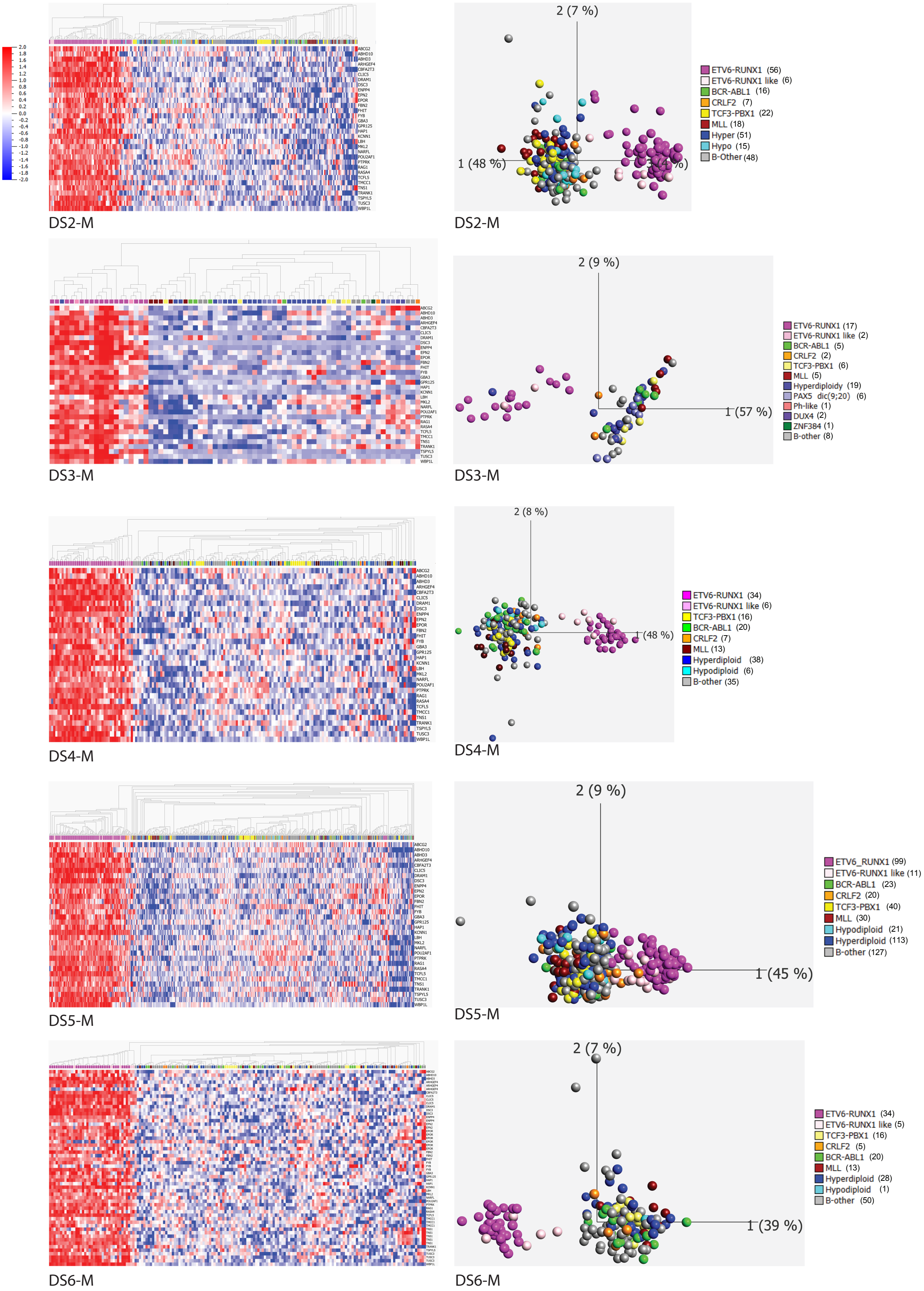

Figure S3. Heatmaps and PCA plots produced using hierarchical clustering analysis of the *RAG1*-signature genes in DS2-6-M. In pink are represented those B-other samples that cluster together with ER samples. These B-other samples are therefore named ER-like.
