# Supplementary material for: RAG1 co‐expression signature identifies ETV6‐RUNX1‐like B‐cell precursor acute lymphoblastic leukemia in children
Source: Cancer Med. 2021 May 13;10(12):3997–4003. doi: 10.1002/cam4.3928 (PMC8209579; doi:10.1002/cam4.3928)
Supplement: Supplementary file 4 — Figure S4 [file CAM4-10-3997-s003.pdf]

# Figure S4

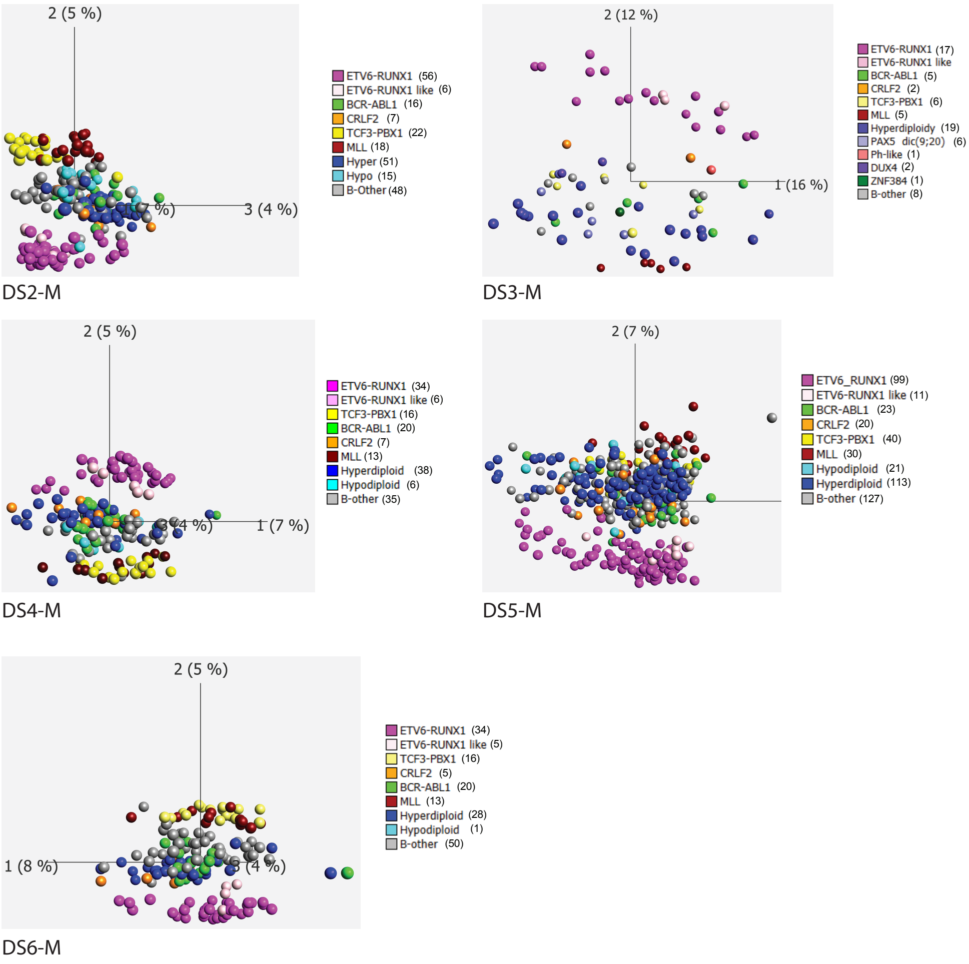


Figure S4. Unsupervised PCA analyses show ER and ER-like BCP-ALL sharing similar gene expression profile in DS2-6-M.
