# Supplementary material for: RAG1 co‐expression signature identifies ETV6‐RUNX1‐like B‐cell precursor acute lymphoblastic leukemia in children
Source: Cancer Med. 2021 May 13;10(12):3997–4003. doi: 10.1002/cam4.3928 (PMC8209579; doi:10.1002/cam4.3928)
Supplement: Supplementary file 5 — Figure S5 [file CAM4-10-3997-s007.pdf]

Figure S5

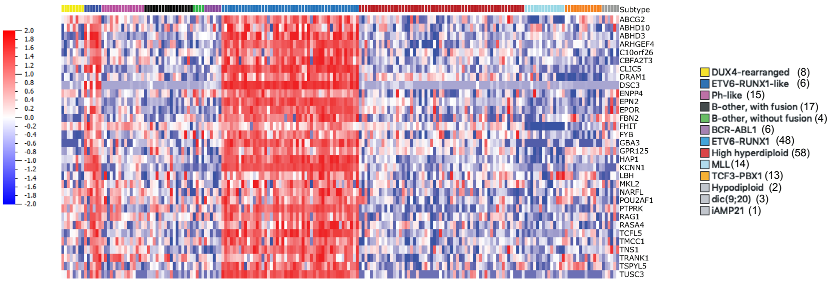

DS8-R

Figure S5. Heatmap produced using the *RAG1*-signature genes in DS8-R.
Note that the color code assigned to the BCP-ALL subtypes is different from the previous figures.
