# Supplementary material for: RAG1 co‐expression signature identifies ETV6‐RUNX1‐like B‐cell precursor acute lymphoblastic leukemia in children
Source: Cancer Med. 2021 May 13;10(12):3997–4003. doi: 10.1002/cam4.3928 (PMC8209579; doi:10.1002/cam4.3928)
Supplement: Supplementary file 8 — Figure S8 [file CAM4-10-3997-s002.pdf]

Figure S8

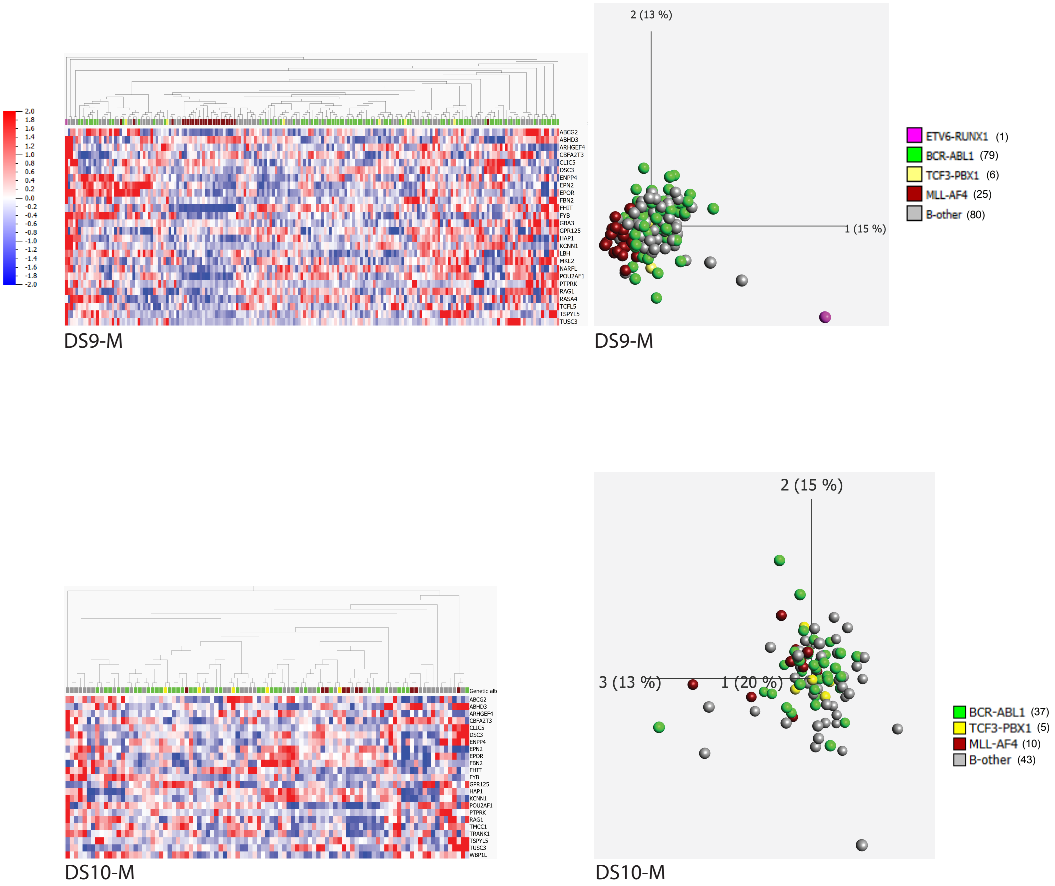

Figure S8. Heatmaps and PCA plots produced using hierarchical clustering analysis of the *RAG1*-signature genes in DS9-10-M. These datasets comprise samples from adult patients.
