# Supplementary material for: RAG1 co‐expression signature identifies ETV6‐RUNX1‐like B‐cell precursor acute lymphoblastic leukemia in children
Source: Cancer Med. 2021 May 13;10(12):3997–4003. doi: 10.1002/cam4.3928 (PMC8209579; doi:10.1002/cam4.3928)
Supplement: Supplementary file 9 — Table S1‐S2 and S4 [file CAM4-10-3997-s006.doc]

**Table S1.** Genetic subtypes in data sets used

|  | DS1-M | DS2-M | DS3-M | DS4-M | DS5-M | DS6-M | DS7-R | DS8-R | DS9-M | DS10-M |
| --- | --- | --- | --- | --- | --- | --- | --- | --- | --- | --- |
| ETV6-RUNX1 | 23 | 56 | 17 | 34 | 99 | 34 | 18 | 48 | 1 | 0 |
| ETV6-RUNX1 like | 4 | 6 | 2 | 6 | 11 | 5 | 4 | 6 | 0 | 0 |
| TCF3-PBX1 | 8 | 22 | 6 | 16 | 40 | 16 | 0 | 13 | 6 | 5 |
| BCR-ABL1 | 18 | 16 | 5 | 20 | 23 | 20 | 6 | 6 | 79 | 37 |
| CRLF2 | 11 | 7 | 2 | 7 | 20 | 5 | 5 | 0 | 0 | 0 |
| MLL | 15 | 18 | 5 | 13 | 30 | 13 | 7 | 14 | 25 | 10 |
| Hyperdiploidy | 27 | 51 | 20 | 38 | 113 | 28 | 42 | 58 | 0 | 0 |
| Hypodiploidy | 1 | 15 | 0 | 6 | 21 | 1 | 0 | 2 | 0 | 0 |
| ERG | 11 | 0 | 0 | 0 | 0 | 0 | 0 | 0 | 0 | 0 |
| ZNF384 | 0 | 0 | 1 | 0 | 0 | 0 | 6 | 0 | 0 | 0 |
| BCR-ABL1 like | 0 | 0 | 1 | 0 | 0 | 0 | 0 | 15 | 0 | 0 |
| PAX5 dic(9;20) | 0 | 0 | 6 | 0 | 0 | 0 | 6 | 3 | 0 | 0 |
| DUX4 | 0 | 0 | 2 | 0 | 0 | 0 | 9 | 8 | 0 | 0 |
| B-other | 9 | 48 | 8 | 35 | 127 | 50 | 13 | 22 | 80 | 43 |
| Total | 127 | 239 | 75 | 175 | 484 | 172 | 116 | 195 | 191 | 95 |

**Table S2.** RAG1-signature gene list

| # | Genes | PCC^#^ value (%) |
| --- | --- | --- |
| 1 | *ABCG2* | *60.00* |
| 2 | *ABHD10* | *60.12* |
| 3 | *ABHD3* | *61.21* |
| 4 | *ARHGEF4* | *60.05* |
| 5 | *CBFA2T3* | *63.26* |
| 6 | *CLIC5* | *76.00* |
| 7 | *DRAM1* | *62.00* |
| 8 | *DSC3* | *65.58* |
| 9 | *ENPP4* | *60.26* |
| 10 | *EPN2* | *60.76* |
| 11 | *EPOR* | *65.00* |
| 12 | *FBN2* | *65.6* |
| 13 | *FHIT* | *71.53* |
| 14 | *FYB* | *60.45* |
| 15 | *GBA3** | *61.37* |
| 16 | *GPR125 (ADGRA3)* | *60.59* |
| 17 | *HAP1* | *63.08* |
| 18 | *KCNN1* | *63.89* |
| 19 | *LBH* | *62.58* |
| 20 | *MKL2* | *61.25* |
| 21 | *NARFL* | *68.71* |
| 22 | *POU2AF1* | *66.94* |
| 23 | *PTPRK* | *66.29* |
| 24 | ***RAG1*** | ***100.00*** |
| 25 | *RASA4* | *62.52* |
| 26 | *TCFL5* | *67.49* |
| 27 | *TMCC1* | *61.31* |
| 28 | *TNS1* | *64.09* |
| 29 | *TRANK1* | *60.13* |
| 30 | *TSPYL5* | *61.39* |
| 31 | *TUSC3* | *60.02* |
| 32 | *WBP1L (C10orf26)* | *73.11* |

* not found in DS7-R; ^#^ Pearson Co-efficiency Correlation in DS1-M

**Table S3.** RAG-1 signature and RAG-2 gene expression levels, see separate excel file

**Table S4.** Frequencies of ER-like BCP-ALL expressing the RAG1-signature genes

| GEO  accession | Data sets | Total  Patient # | B-other | RAG1-signature ER-like,  n (% of total) |
| --- | --- | --- | --- | --- |
| GSE26281 | DS1 | 127 | 13 | 4 (3.1) |
| GSE28497 | DS2 | 239 | 54 | 7 (2.9) |
| GSE47051 | DS3 | 75 | 11 | 3 (4.0) |
| GSE12995 | DS4 | 175 | 40 | 5 (2.9) |
| GSE33315 | DS5 | 484 | 136 | 9 (1.9) |
| GSE26366 | DS6 | 172 | 53 | 5 (2.9) |
| RNA-seq1 | DS7 | 116 | 17 | 4 (3.4) |
| RNA-seq2 | DS8 | 195 | 50 | 6 (3.1) |
|  | Total | 1583 | 374 | 43 (2.7) |

**References**

1. Lee ST, Xiao Y, Muench MO, Xiao J, Fomin ME, Wiencke JK, Zheng S, Dou X, de Smith A, Chokkalingam A, Buffler P, Ma X, et al. A global DNA methylation and gene expression analysis of early human B-cell development reveals a demethylation signature and transcription factor network. *Nucleic Acids Res* 2012;**40**: 11339-51.

2. Figueroa ME, Chen SC, Andersson AK, Phillips LA, Li Y, Sotzen J, Kundu M, Downing JR, Melnick A, Mullighan CG. Integrated genetic and epigenetic analysis of childhood acute lymphoblastic leukemia. *J Clin Invest* 2013;**123**: 3099-111.

3. Coustan-Smith E, Song G, Clark C, Key L, Liu P, Mehrpooya M, Stow P, Su X, Shurtleff S, Pui CH, Downing JR, Campana D. New markers for minimal residual disease detection in acute lymphoblastic leukemia. *Blood* 2011;**117**: 6267-76.

4. Nordlund J, Backlin CL, Wahlberg P, Busche S, Berglund EC, Eloranta ML, Flaegstad T, Forestier E, Frost BM, Harila-Saari A, Heyman M, Jonsson OG, et al. Genome-wide signatures of differential DNA methylation in pediatric acute lymphoblastic leukemia. *Genome Biol* 2013;**14**: r105.

5. Mullighan CG, Su X, Zhang J, Radtke I, Phillips LA, Miller CB, Ma J, Liu W, Cheng C, Schulman BA. Deletion of IKZF1 and prognosis in acute lymphoblastic leukemia. *New England Journal of Medicine* 2009;**360**: 470-80.

6. Zhang J, Ding L, Holmfeldt L, Wu G, Heatley SL, Payne-Turner D, Easton J, Chen X, Wang J, Rusch M, Lu C, Chen SC, et al. The genetic basis of early T-cell precursor acute lymphoblastic leukaemia. *Nature* 2012;**481**: 157-63.

7. Kannan S, Fang W, Song G, Mullighan CG, Hammitt R, McMurray J, Zweidler-McKay PA. Notch/HES1-mediated PARP1 activation: a cell type-specific mechanism for tumor suppression. *Blood* 2011;**117**: 2891-900.

8. Marincevic-Zuniga Y, Dahlberg J, Nilsson S, Raine A, Nystedt S, Lindqvist CM, Berglund EC, Abrahamsson J, Cavelier L, Forestier E, Heyman M, Lonnerholm G, et al. Transcriptome sequencing in pediatric acute lymphoblastic leukemia identifies fusion genes associated with distinct DNA methylation profiles. *J Hematol Oncol* 2017;**10**: 148.

9. Lilljebjorn H, Henningsson R, Hyrenius-Wittsten A, Olsson L, Orsmark-Pietras C, von Palffy S, Askmyr M, Rissler M, Schrappe M, Cario G, Castor A, Pronk CJ, et al. Identification of ETV6-RUNX1-like and DUX4-rearranged subtypes in paediatric B-cell precursor acute lymphoblastic leukaemia. *Nat Commun* 2016;**7**: 11790.

10. Geng H, Brennan S, Milne TA, Chen WY, Li Y, Hurtz C, Kweon SM, Zickl L, Shojaee S, Neuberg D, Huang C, Biswas D, et al. Integrative epigenomic analysis identifies biomarkers and therapeutic targets in adult B-acute lymphoblastic leukemia. *Cancer Discov* 2012;**2**: 1004-23.

11. Chiaretti S, Li X, Gentleman R, Vitale A, Wang KS, Mandelli F, Foa R, Ritz J. Gene expression profiles of B-lineage adult acute lymphocytic leukemia reveal genetic patterns that identify lineage derivation and distinct mechanisms of transformation. *Clin Cancer Res* 2005;**11**: 7209-19.
